# Prognostic CMR parameters for heart failure and arrhythmias in large cohort of well treated thalssemia major patients

**DOI:** 10.1186/1532-429X-16-S1-O33

**Published:** 2014-01-16

**Authors:** Antonella Meloni, Vincenzo Positano, Giuseppe Rossi, Petra Keilberg, Silvia Macchi, Stefano Pulini, Gianluca Valeri, Paolo Preziosi, Cristina Salvatori, Massimo Lombardi, Alessia Pepe

**Affiliations:** 1 CMR Unit, Fondazione G.Monasterio CNR-Regione Toscana and Institute of Clinical Physiology, Pisa, Italy; 2 Epidemiology and Biostatistics Unit, Institute of Clinical Physiology, CNR, Pisa, Italy; 3 Servizio Trasfusionale, Ospedale Santa Maria delle Croci, Ravenna, Italy; 4 U.O. Ematologia Clinica, Osped. Civile "Spirito Santo", Pescara, Italy; 5 Dipartimento di Radiologia, Azienda Ospedaliero-Universitaria Ospedali Riuniti "Umberto I-Lancisi-Salesi", Ancona, Italy; 6 U.O.C. Diagnostica per Immagini e Interventistica, Policlinico "Casilino", Roma, Italy

## Background

Cardiac complications are the main cause of death in thalassemia major (TM) patients. Cardiovascular Magnetic Resonance (CMR) plays a key role in their management, assessing myocardial iron overload (MIO), biventricular function, atrial dimensions, and myocardial fibrosis. We evaluated the predictive value of CMR parameters for heart failure and arrhythmias.

## Methods

We followed prospectively 487 TM patients free of a cardiac complications at the first CMR. All prognostic variables associated with the outcome at the univariate Cox model were placed in the multivariate model and were ruled out if they did not significantly improve the adjustment.

## Results

**Figure 1:**
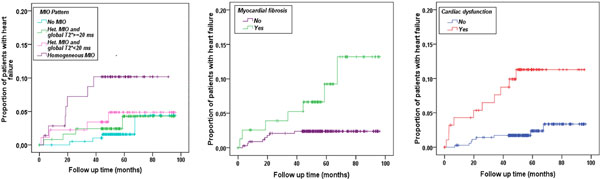


**Figure 2:**
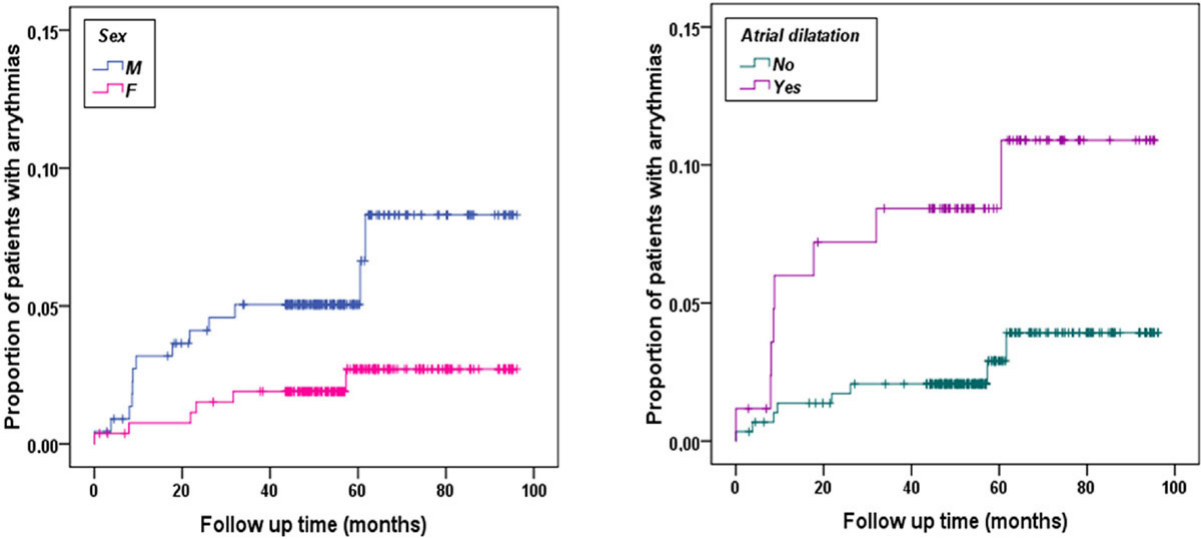


At baseline the mean age was 29.5 ± 9.0 years and 222 patients were males. The mean follow-up time was 58 ± 18 months. After the first CMR only the 37.8% of the patients did not change the chelation regimen or the frequency/dosage. We recorded 19 episodes of heart failure. Male sex, heart iron, ventricular dysfunction, ventricular dilation, atrial dilation, and myocardial fibrosis were significant univariate prognosticators. In the multivariate analysis the independent predictive factors were an homogeneous pattern of MIO (compared to no MIO) (HR = 5.81, 95%CI = 1.42-23.74, P = 0.014), myocardial fibrosis (HR = 4.93, 95%CI = 1.71-14.71, P = 0.003) and ventricular dysfunction (HR = 3.45, 95%CI = 1.19-9.98, P = 0.022). Figure 1 shows the Kaplan-Meier survival curves for the prognosticators. Arrhythmias (all supraventicular hyperhyperkinetic) occurred in 19 patients. Male sex, atrial dilatation and ventricular dysfunction were significant univariate prognosticators. In the multivariate analysis the independent predictive factors were male sex (HR = 3.17, 95%CI = 1.02-9.87, P = 0.047) and atrial dilation (HR = 3.07, 95%CI = 1.14-8.23, P = 0.026). Figure 2 shows the Kaplan-Meier survival curves for the prognosticators. Serum ferritin and liver iron were not predictive factors for heart failure or arrhythmias.

## Conclusions

We detected few cardiac events thanks to a MR-guided, patient-specific adjustment of the chelation therapy. Severe and homogeneous MIO, myocardial fibrosis and ventricular dysfunction identify patients at high risk of heart failure. Heart T2* doesn't have any power in predicting arrhythmias while male sex and atrial dilation are independent prognosticators.

## Funding

The MIOT project receives "no-profit support" from industrial sponsorships (Chiesi and Apopharma). This study was also supported by: "Ministero della Salute, fondi ex art. 12 D.Lgs. 502/92 e s.m.i., ricerca sanitaria finalizzata anno 2006" e "Fondazione L. Giambrone".

